# Outcome of active TB case finding among a nomadic population in Nigeria

**DOI:** 10.5588/pha.25.0003

**Published:** 2025-06-04

**Authors:** S. Gande, C. Ogbudebe, B. Odume, O. Chukwuogo, J. Emefieh, A. Babayi, A. Yakubu, S. John, S. Abdulkarim

**Affiliations:** ^1^ KNCV Tuberculosis Foundation Nigeria, Abuja, Nigeria;; ^2^ Janna Health Foundation, Yola, Adamawa State, Nigeria.

**Keywords:** public health facilities, presumptive TB, TB diagnosis, Nomads

## Abstract

**SETTING:**

Finding TB among nomads, a high-risk group, can help address the global challenge of missing TB cases. Nigeria accounts for 6.2% of the 3.1 million missing TB cases.

**OBJECTIVE:**

To describe the outcome of active TB case-finding (ACF) among nomads in Bauchi State, northeastern Nigeria.

**DESIGN:**

KNCV implemented ACF in eight Local Government Areas of Bauchi State from June 2020 to December 2023. Trained community volunteers screened nomads and ensured diagnostic testing of sputum samples of nomads with presumptive TB. Community volunteers helped to link those with confirmed TB to treatment.

**RESULTS:**

We screened 43,070 nomads (35,533 adults; 7,537 children) of which 43% were males. Presumptive TB was detected among 12,387, of whom 850 (6.9%) had active TB, including 61 children. Overall, 99% of people with active TB were put on treatment. Presumptive TB yield was higher among the nomads, but active TB yield was comparable with the general population. Nomads contributed 5% to the total number of people notified with TB from Bauchi State.

**CONCLUSION:**

ACF among nomads resulted in improved TB notification and can help improve the identification of missing TB cases in Nigeria.

Of the 3.1 million gap between estimated incident TB cases and those newly notified (‘missing TB cases’), 6.2% is accounted for by the TB situation in Nigeria.^1^ This challenge of ‘missing TB cases’ implicates sub-optimal TB case detection. Traditionally, passive TB case finding has been the practice in most low- to middle-income countries (LMICs), such as Nigeria.^2,3^ However, the consistently high figures for ‘missing’ TB cases have been attributed to the insufficiency of passive TB case-finding.^4,5^ To address this, active TB case finding (ACF) is an important complementary strategy,^5^ which benefits certain TB high-risk groups such as people with HIV, diabetes, and other immunocompromised conditions, the homeless, imprisoned, refugees and migrants.^6^ Nomadic populations, characterized by their seasonal migration for game or in search of pasture for their animals have been implicated in spreading diseases.^7^ Nomads, especially pastoral nomads, are at high risk of TB due to poor access to health services and health-seeking behaviour resulting from their cyclical movement.^7^ Other factors include overcrowding and unpasteurized milk consumption. Demonstrating the burden of TB among nomads, a study in Mali reported that half of the 50 nomads presumed to have TB were diagnosed with active TB.^8^ Another study in Kenya showed a TB case detection rate of 176/100,000 among nomads compared to 61/100,000 nationally.^9^

There are relatively few studies on TB case detection among nomads in Nigeria. A 15-month community-based ACF in three northeast states (Adamawa, Gombe, and Taraba) led to a 14% increase in the total TB notifications from these states.^10^ Similarly, a two-year ACF Adamawa state increased total TB notification by 24.5%.^11^ Here, we report on the outcome of an ACF intervention to improve TB case notification among nomads in Bauchi State, northeast Nigeria.

## METHODOLOGY

### Location

The ACF intervention was carried out in eight of the 20 Local Government Areas (LGAs) of Bauchi, one of six North Eastern States of Nigeria. Bauchi shares a border with the States of Jigawa, Kano, Kaduna, Plateau, Taraba, Gombe, and Yobe (Figure 1).^12^ Bauchi State is home to more than 55 ethnic groups, including the Fulani, which is the major nomadic population in the state.^13^

**FIGURE.**
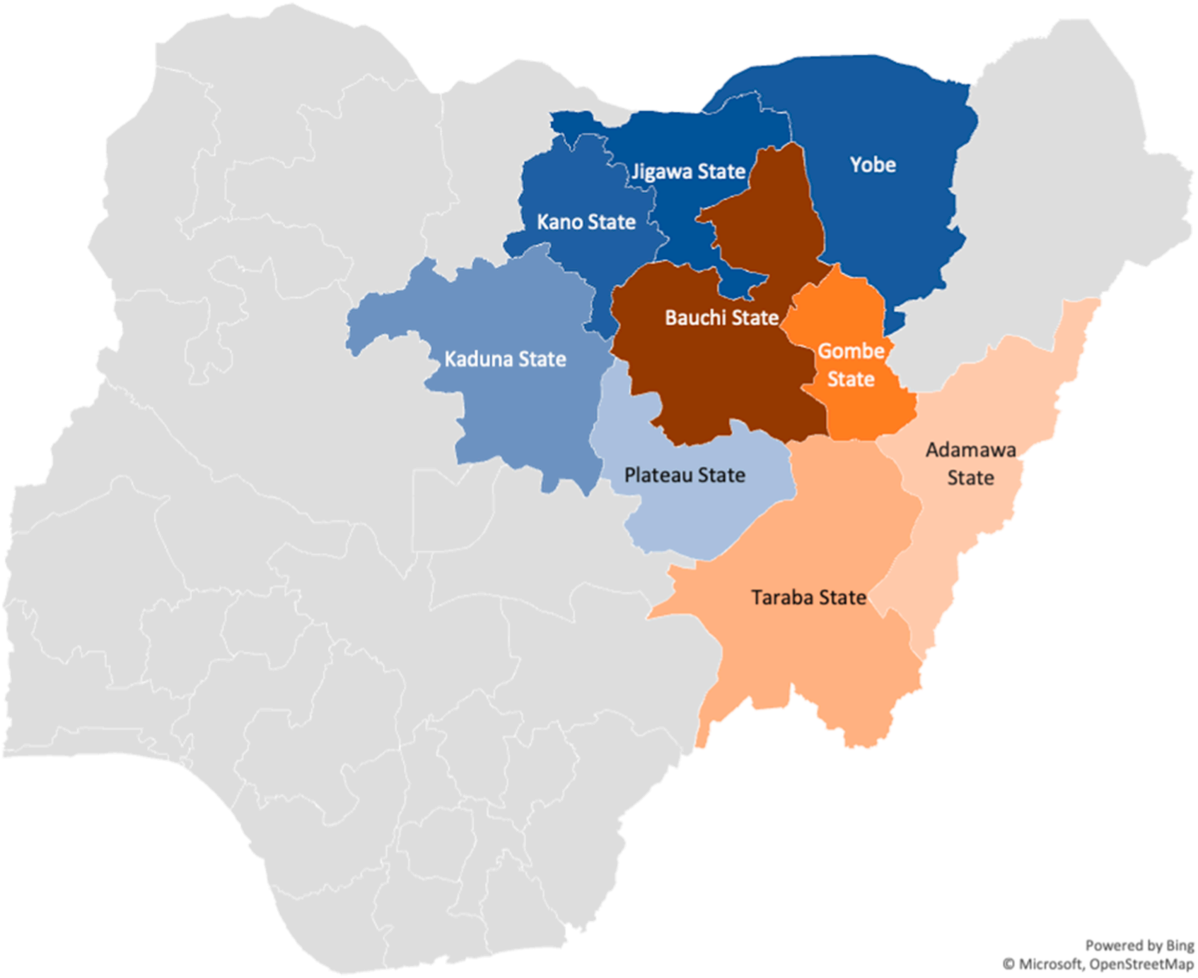
Map of Bauchi State and other States with reports on TB among nomads and bordering States. Border States = blue; States with reports on TB among nomads = orange; Study State = brown.

### Intervention

In 2020, KNCV in collaboration with the Janna Health Foundation (JHF), a community-based organization experienced in TB programming among nomads,^10^ initiated an ACF intervention among nomadic populations in Bauchi State using a quasi-experimental or observational cohort study design was conducted. The ACF intervention involved symptomatic TB screening, transportation of sputum from nomads with presumptive TB to health facilities with TB diagnostic capacity for confirmation of active TB, linkage of nomads detected with TB to directly observed treatment shortcourse (DOTS) centers and notification of all confirmed nomads with TB to the National TB and Leprosy Control Programme (NTBLCP).

### Mapping of nomadic settlements and community engagement

Maps for livestock management in Bauchi State were used to identify nomadic settlements and communities and determine the ACF intervention sites. We charted all cattle routes and health facilities including TB DOTS facilities and TB diagnostic centers closest to migration routes. The mapping was conducted in collaboration with the Bauchi State Ministry of Agriculture and Rural Development and the Janna Foundation between April–June 2020. Together with the Bauchi State Ministry of Health, service awareness, and promotion campaigns were conducted using jingles disseminated through local radio and television stations in Fulfulde and Hausa languages (popular spoken languages among the nomads) before and continuously over the implementation period of the intervention. These campaigns included awareness about TB-related symptoms and the availability of free diagnosis and treatment for identified cases. Community engagement (CE) meetings were held with community gatekeepers (chiefs of nomadic settlements, market chairmen, and heads of nomadic schools) to inform design and promote acceptability of the ACF intervention. From each nomadic community, volunteers were identified, trained and assigned specific responsibilities.

### Presumptive TB screening process

Before the screening, all community volunteers, staff at DOTS and TB diagnostic centers, and LGA TB supervisors were trained (or retrained) on the principles of ACF, presumptive TB screening, diagnostic referral, and linkage to care at designated DOTS centers. Training was conducted centrally in the State over five days, and continually on the job. TB screening sessions began with brief health education messages about TB in Hausa or Fulfulde. Screening was voluntary with verbal consent obtained per routine clinical care from interested persons. The screening was conducted by community volunteers supported by the LGA TB supervisors using a basic questionnaire to assess for TB symptoms per the WHO consolidated guidelines on TB.^14^ The symptoms screened for include a prolonged cough lasting 2 weeks or more and any other symptoms compatible with TB such as cough of any duration, hemoptysis, weight loss, fever, and night sweats. Symptom assessment tools was rigorously validated to ensure reliability and accuracy in TB case detection. This validation process involved the inclusion of cardinal TB symptoms (cough ≥2 weeks, fever, weight loss, and night sweats) in line with the WHO guideline.

### TB evaluation and linkage to care

People with presumptive TB submitted sputum specimens for diagnostic testing with Gene Xpert assay or TB-LAMP assays per the National TB Policy.^15^ The specimens were collected and transported by community volunteers to 1 of the 12 TB diagnostic centers most proximal to the community of the nomads using standard procedure.^16^ For children, symptomatic TB screening was done by the LGA TB supervisor. Children with symptoms of TB and their parents or guardians were batched and transported to the nearest diagnostic center for sputum collection and diagnostic testing. Chest X-ray was used instead of Xpert assay for children unable to produce sputum. The LGA TB supervisors retrieved TB diagnostic test results to actively link eligible individuals to a DOTS center closest to their communities. At the DOTS centers, all newly diagnosed TB patients received treatment support and monitoring from community volunteers and local leaders. All confirmed people with TB were notified following the National TB guidelines.

### Data collection

Data on the TB cascade was collected using tools from the National TB Program. Nomadic cases were identified with the tag ‘nomads’ in the comment section of the tools. Trained community volunteers collected data at the community level, and recorded screening and diagnostic testing outcomes. Data clerks at the DOTS centers recorded treatment linkage and initiation. De-identified data needed for this program was abstracted from the National tools onto the TB Commcare, an application used by the KNCV for collecting TB-related data. Data quality assurance and validation were conducted during weekly data quality review meetings at the facilities, monthly meetings of the KNCV M&E staff, and quarterly TB cohort review meetings at the LGA and State levels. Historical and recent TB data from the State was retrieved from the TB Register at each Local Government to allow comparison between nomadic and general population TB case notifications.

### Data analysis

We descriptively analyzed data on the number of persons screened for TB, people with presumptive TB identified, evaluated, diagnosed and enrolled on treatment. We compared notifications from the ACF intervention against the overall notifications from the general population in Bauchi state. All TB patients diagnosed and notified among nomads during the intervention period were included in our analysis. Information on age, sex, laboratory results, and TB treatment initiation was extracted using standardized definitions by the WHO and the International Union Against Tuberculosis and Lung Disease. All data analysis was done in Microsoft® Excel.

We obtained administrative approval for the ACF intervention from the Ministries of Health and Agriculture and Rural Development in Bauchi State. The analysis reported here was determined as a non-research program evaluation because it required no direct contact with human subjects (no interview or sample collection), and only de-identified pooled program data that formed part of the standard of care were used. Written informed consent was not required.

## RESULTS

Overall, 1,083 nomadic settlements were mapped of which 719 were confirmed to be within the boundaries of Bauchi State and the 8 LGAs for our ACF intervention. There were also 78 DOTS centers accessible to the nomadic communities, with an average of one DOTS center to nine nomadic communities. The farthest distance between a nomadic community to a DOTS center was 334 km and the shortest was 1 km. We screened 43,070 nomads (35,533 adults; 7,537 children), of which 43.0% were males and 57.0% were females. Of the total nomads screened, 12,387 were determined to have presumptive TB. Of the presumptive TB cases, 850 (6.9%) were confirmed to have active TB, out of which 690 (81.2%) were bacteriologically confirmed and 160 (18.8%) were clinically confirmed, (chest x-ray and symptoms). Of the confirmed people with TB, 849 (99.9%) were started on appropriate TB treatment. Children (0–14 years) accounted for 61 (7.2%) of the confirmed people with TB, and all were commenced on appropriate TB treatment (Table 1). Among the children confirmed with TB, 34% (21/61) were clinically diagnosed against 17.6% (139/789) among adults. Among adults, women were diagnosed with more TB (67.7%) compared to men (32.3%) (534 versus 255). This trend was similar among children with 34 TB cases among female children (55.7%) versus 27 TB cases among male children (44.3%). The age disaggregation of the TB cascade shows that adults between 25–54 years had the highest number of TB cases, while the least was among the 65+ age group.

**TABLE 1.** Demographic characteristics of nomads by TB cascade, Bauchi State, North East Nigeria.

		Clients screened for TB	Clients with presumptive TB	Presumptive cases evaluated for TB	Diagnosed with All forms of TB	Bac+ TB patients	Clinically diagnosed TB	Childhood TB	Started on TB treatment
Total		43,070	12,387	11,848	850	690	160	61	849
Sex	Male	18694	4770	4589	282	237	45	27	282
	Female	24376	7617	7259	568	453	115	34	567
Age group (years)									
	0–4	3,117	479	431	19	10	9	19	19
	5–14	4,420	950	882	42	30	12	42	42
	15–24	5,436	1,663	1,602	82	64	17	0	82
	25–34	6,103	2,077	2,018	176	156	20	0	177
	35–44	6,482	2,259	2,175	191	159	33	0	191
	45–54	6,497	2,035	1,973	178	152	27	0	177
	55–64	6,113	1,732	1,639	106	86	19	0	106
	65+	4,902	1,192	1,128	56	33	23	0	55

We compared the TB burden between nomads in our study and the general population from 2020–2023. TB presumptive yield was significantly higher among nomads compared to the general population. Among the nomads, the highest TB presumptive yield was 86.6% in 2020, which decreased steadily to 20.1% in 2023. TB presumptive yield for the general population ranged between 6.5% in 2021 and 8.2% in 2023. Actual TB yield following molecular diagnostic testing was comparable between nomads and the general population. The highest TB yield for the nomadic and the general population was 9.4% and 9.6% in 2023 respectively (Table 2), with a 7% overall yield in 2020-2023. The highest contribution of the nomadic populations to the TB cascade in Bauchi State was in 2020 (10.3%). However, for subsequent years, (2021–2023), the contribution of the nomadic population to all people with TB and those put on treatment was between 4% to 5% (Table 2). Cumulatively, the nomadic populations contributed 5% to the TB cases notified from Bauchi state from 2020-2023. The nomads recorded zero pre-treatment loss to follow-up, except in 2021 when it was 0.6%. While among the general population, it was highest in 2020 (1.5%) but declined steadily to 0.3% by December 2023 (Table 2).

**TABLE 2.** Details of the TB ACF cascade and efficiency among nomads and the general population, Bauchi State, North East Nigeria (2020–2023).

	2020		2021		2022		2023	
	Nomads	Non-Nomads	Nomads	Non-Nomads	Nomads	Non-Nomads	Nomads	Non-Nomads
	n (%)	n (%)	n (%)	n (%)	n (%)	n (%)	n (%)	n (%)
Clients screened for TB	4,888	155682	6406	942482	13795	962326	17981	813345 (6.2)
Presumed to have TB	2016 (41)	12741 (8.2)	2648 (41.3)	61424 (29.8)	4104 (29.8)	66500 (9.3)	3619 (20.1)	66816 (98.4)
Evaluated for TB	1765 (86)	11348 (89.1)	2486 (93.9)	59037 (99.3)	4075 (99.3)	64395 (96.8)	3522 (97.3)	65764 (98.4)
Diagnosed with TB	103 (6)	913 (8.1)	168 (6.8)	3376 (5.7)	248 (2.1)	5397 (8.4)	331 (9.4)	6309 (9.6)
Bacteriologically diagnosed TB patients	86 (84)	739 (80.9)	104 (61.9)	2660 (78.8)	196 (79)	4521 (83.8)	304 (91.8)	4512 (71.5)
Clinically diagnosed	17 (17)	174 (19.10)	64 (38.1)	716 (21.2)	52 (21)	876 (16.2)	27 (8.2)	1797 (28.5)
Childhood TB	2 (3)	65 (7.10)	12 (7.1)	262 (7.8)	18 (7.3)	362 (6.7)	29 (8.8)	475 (7.5)
Started on TB treatment	103 (100)	899 (98.40)	167	3348 (99.2)	248 (100)	5333 (98.8)	331 (100)	6288 (99.7)
Presumptive yield (%)	86.60%	7.70%		6.50%	29.70%	6.90%	20.10%	8.20%
Evaluation rate (%)	87.50%	89.10%		96.10%	99.30%	96.80%	97.30%	98.40%
TB Yield (%)	5.8	8.00%		5.70%	6.10%	8.40%	9.40%	9.60%
Pre-treatment loss to follow-up (%)	0%	1.50%		0.80%	0%	1.20%	0%	0.30%
Contribution to total notifications	10.30%				4.20%		5%	
NNS*	47	171	38	279	56	178	54	129
NNT**	17	12	15	17	16	12	11	10

* NNS = number needed to screen. Clients screened versus clients diagnosed with TB.

** NNT = number needed to test. Clients evaluated for TB versus clients diagnosed with TB.

The ACF among nomads had much lower Number Needed to Screen (NNS) compared to the general population in the study period. The Number Needed to Test (NNT) was comparable but slightly higher among the nomads compared with the general population over the study period (NNT 14 versus NNS 13), but the difference varied for each year (Table 2).

## DISCUSSION

With the ACF intervention, we identified 850 new people with TB between 2020 and 2023. The intervention successfully contributed 5% to the total new active TB cases identified in the State within this period, despite nomads constituting only 2% of the persons screened for TB. We adopted other complementary strategies in our ACF, such as the use of community volunteers to help with community identification and presumptive TB screening, transportation of sputum samples to decentralized diagnostic facilities, retrieval of results, and collaboration with the LGA TB supervisors to ensure that persons with confirmed TB were commenced on appropriate treatment. This combination of approaches helped shift the burden of seeking care for TB to the system, and away from the patient, thereby significantly reducing loss of wages and out-of-pocket expenditure for patients.^17^ The predominance of molecular TB diagnostics in our intervention is reported to improve case detection and finding missing people with TB.^18^ The high presumptive TB yield, linkage to DOTS which was at least 99%, and modest NNT (14) recorded in our intervention may be attributed to the success of these combination strategies. Similar outcomes have been reported in other projects implementing these combination strategies.^11,19,20^

Nigeria’s TB Strategic Plan 2021–2025 targeted identifying 4,234, 4,354, and 4,477 TB cases from nomadic populations in 2021, 2022 and 2023 respectively from 12 focal states including Bauchi.^21^ Our intervention in only 8 LGAs out of 20 in Bauchi contributed 4.0%, 5.7%, and 7.4% to these national targets respectively. While this is an important contribution, it could be optimized with a State-wide adoption of the strategy across all nomadic populations. The 7% TB case detection we found is less than the 11% reported in a similar study in Adamawa State,^11^ but slightly higher than the rate (5.4%) reported in the JHF case study.^10^ The variation may be due to differences in nomadic populations across States, or the influence of different TB infection risk drivers on nomadic populations. Migration has been established as an important factor in the distribution of diseases^22^ and higher TB rates have been reported among migrant populations.^23,24^ In LMICs like Nigeria, trans-border and internal migration of the people are said to impact on national TB burden even more than in the case of migration from high TB burden LMICs to low TB burden high-income countries.^25^

Contrary to global and country-specific reports,^26^ we found more TB among women than men. Lower TB rates among women have been attributed to underreporting due to various factors such as isolation, paternalism in healthcare, and other decision-making as well as ownership of financial resources.^26,27^ Additionally, due to differential healthcare access and bias associated with testing, women have better healthcare access unlike Nomadic men who’s mobility limits healthcare access. They often travel for trade, herding or seasonal labor. Thus, reducing their visits to healthcare. The predominance of passive TB case finding in LMICs also contributes to lower TB identification among women.^28^ Thus, the higher TB among women in our intervention may be due to the ACF strategy deployed. However, more studies to confirm the effect of passive TB case-finding on the underreporting of TB among women in general and nomadic populations are needed.^28^

Our study had some limitations. Focusing on only 8 out of 20 LGAs in Bauchi State may have excluded other nomadic populations in the State. However, this is unlikely to have affected our coverage of nomadic populations because the selection of the LGAs was based on the density of nomadic populations in the State. We did not disaggregate the outcome data by nomadic communities and screening locations, hence our inability to estimate location-specific efficiencies that could have benefitted future interventions in Bauchi or other States. Despite these limitations, our study has demonstrated the important contributions that TB ACF can make toward identifying missing people with TB among high-risk populations, as nomads.

## CONCLUSION

Our study has shown that ACF for TB in migrant communities, such as the nomads in Nigeria, results in improved detection of people with TB and has the potential to narrow the TB case detection gap if implemented at scale.
